# Draft Genome Sequences of *Staphylococcus* Podophages JBug18, Pike, Pontiff, and Pabna

**DOI:** 10.1128/MRA.00054-19

**Published:** 2019-02-21

**Authors:** Emma K. Culbertson, S. M. Nayeemul Bari, Vidya Sree Dandu, Jennie M. Kriznik, Samuel E. Scopel, Samuel P. Stanley, Kim Lackey, Adriana C. Hernandez, Asma Hatoum-Aslan

**Affiliations:** a Department of Biological Sciences, University of Alabama, Tuscaloosa, Alabama, USA; b Center for Phage Technology, Texas A&M University and Texas A&M AgriLife, College Station, Texas, USA; Queens College

## Abstract

We report here the draft genome sequences of *Staphylococcus* bacteriophages JBug18, Pike, Pontiff, and Pabna, which infect and lyse S. epidermidis and S. aureus strains. All bacteriophages belong to the morphological family *Podoviridae* and constitute attractive candidates for use as whole-phage therapeutics due to their compact genomes and lytic lifestyles.

## ANNOUNCEMENT

Antibiotic-resistant staphylococci remain global priority pathogens for which alternative treatments are urgently needed (1). S. epidermidis and S. aureus are opportunistic pathogens commonly found on human skin and mucus membranes (2, 3). The viruses that infect and lyse these organisms (staphylococcal phages) are potent antimicrobials that are being explored as alternatives to conventional antibiotics (4). All known staphylococcal phages are tailed and belong to the *Caudovirales* order and one of three morphological families, *Myoviridae*, *Siphoviridae*, and *Podoviridae* (5). Over 100 staphylococcal phage sequences are currently available in the NCBI database (https://www.ncbi.nlm.nih.gov/genomes/GenomesGroup.cgi); however, the majority of these (∼70%) are siphophages, which are unsuitable for whole-phage antimicrobial applications due to their temperate lifestyles. Furthermore, of the strictly lytic varieties, *Podoviridae* are the rarest, constituting ∼7% of the sequenced collection. Due to their compact, more defined genomes, podophages have the potential to alleviate safety concerns associated with uncharacterized genes in therapeutic phages (6). In addition, their genomes are amenable to genetic manipulation (7) and thus constitute attractive genetic scaffolds into which additional bactericidal capabilities can be built. Therefore, podophages are important additions to the staphylococcal phage collection. Here, we announce the full-genome sequences of four lytic staphylococcal phages that all belong to the morphological family *Podoviridae*.

Phages were isolated from a sample of wastewater collected at the Hilliard Fletcher Wastewater Treatment Plant in Tuscaloosa, Alabama, on 25 August 2015. The phages were isolated using three different staphylococcal hosts, S. epidermidis LM1680, S. epidermidis RP62a, and S. aureus RN4220. To enrich for phages, wastewater was combined with each host in tryptic soy broth supplemented with 5 mM CaCl_2_ and incubated at 37°C with agitation over 3 consecutive nights exactly as described in reference 8. After the third night, cells were pelleted and the supernatant was passed through a 0.45-μm filter. The supernatant was plated with fresh overnight cultures of each host in a semisolid layer of top agar (0.5 × heart infusion agar) as described in reference 8 to visualize plaques. To purify, individual plaques were picked and replated with their respective hosts 5 times. Phage morphology was determined using transmission electron microscopy at the University of Alabama Optical Analysis Facility as previously described (8).

**FIG 1:**
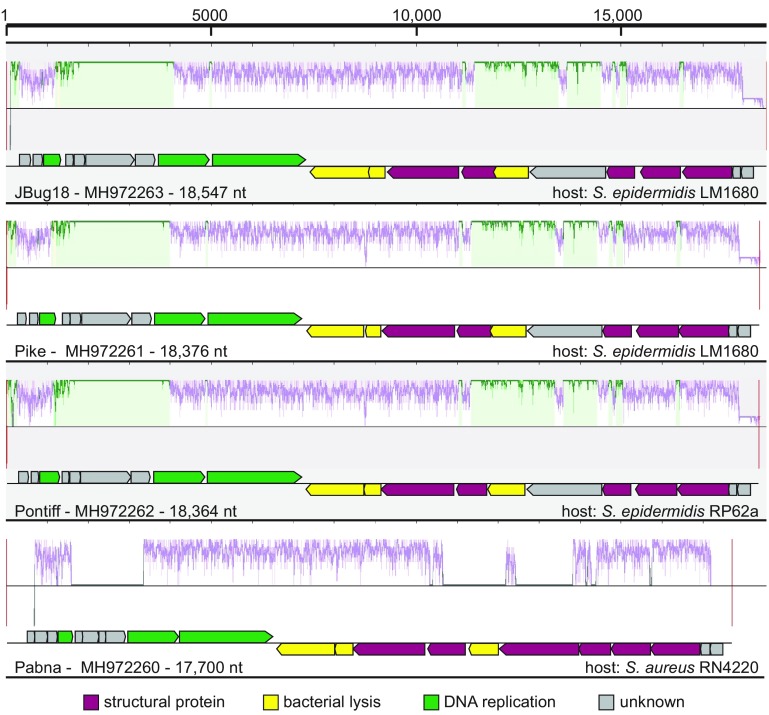
A multiple sequence alignment of staphylococcal phages JBug18, Pike, Pontiff, and Pabna. GenBank accession numbers, genome lengths in nucleotides (nt), and bacterial hosts are indicated for each phage. The alignment was generated using the Mauve software with default settings (http://darlinglab.org/mauve/mauve.html).

Purified phages were propagated and their genomes were extracted using a Promega Wizard DNA cleanup kit (catalog number A7280) in a modified protocol (8). Their DNA was sequenced in an Illumina MiSeq platform using 2 × 250-bp chemistry (paired end) at the Genomic Sequencing and Analysis Facility at the University of Texas in Austin, Texas. Reads were assessed using FastQC v0.11.2 (default settings) and trimmed for quality using the “trim sequence” tool available in the Center for Phage Technology Galaxy instance (https://cpt.tamu.edu/galaxy-pub). Reads of JBug18, Pike, Pontiff, and Pabna were each assembled into a single contig using SPAdes v1.0 with k-mer sizes of 21, 33, and 55. The genomes were assembled into a single contig with depths of coverage of 2559.1, 400.5, 653.6, and 2125.7-fold, respectively, as determined by the following formula: number of reads × read length/contig size. Genes and proteins were predicted using SnapGene v3.1.4 and BLASTp, respectively, both with default settings. All four phages possess genomes with lengths of <20,000 nucleotides and encode 20 to 21 predicted genes (Fig. 1). A multiple sequence alignment reveals that, despite their diverse host ranges, all the phages exhibit a conserved genomic architecture, with two apparent transcriptional units converging near the center. Similar to their hosts, these phages possess a relatively low GC content (29 to 30%).

### Data availability.

The full, annotated genome sequences of JBug18, Pike, Pontiff, and Pabna are available at GenBank under the accession numbers MH972263, MH972261, MH972262, and MH972260, respectively. Raw data can be accessed under the BioSample accession numbers SAMN10713922, SAMN10713923, SAMN10713924, and SAMN10713925, respectively, within BioProject number PRJNA513839.

## References

[1] Tacconelli E, Margrini N 2017 Global priority list of antibiotic-resistant bacteria to guide research, discovery, and development of new antibiotics. World Health Organization, Geneva, Switzerland https://www.who.int/medicines/publications/WHO-PPL-Short_Summary_25Feb-ET_NM_WHO.pdf?ua=1.

[2] Otto M 2009 *Staphylococcus epidermidis*—the ‘accidental’ pathogen. Nat Rev Microbiol 7:555–567. doi:10.1038/nrmicro2182. 19609257 PMC2807625

[3] Peacock SJ, de Silva I, Lowy FD 2001 What determines nasal carriage of *Staphylococcus aureus*? Trends Microbiol 9:605–610. doi:10.1016/S0966-842X(01)02254-5. 11728874

[4] Borysowski J, Lobocka M, Międzybrodzki R, Weber-Dabrowska B, Górski A 2011 Potential of bacteriophages and their lysins in the treatment of MRSA. BioDrugs 25:347–355. doi:10.2165/11595610-000000000-00000. 22050337

[5] Deghorain M, Van Melderen L 2012 The staphylococci phages family: an overview. Viruses 4:3316–3335. doi:10.3390/v4123316. 23342361 PMC3528268

[6] Philipson CW, Voegtly LJ, Lueder MR, Long KA, Rice GK, Frey KG, Biswas B, Cer RZ, Hamilton T, Bishop-Lilly KA 2018 Characterizing phage genomes for therapeutic applications. Viruses 10:188. doi:10.3390/v10040188. PMC5923482 29642590

[7] Bari SMN, Walker FC, Cater K, Aslan B, Hatoum-Aslan A 2017 Strategies for editing virulent staphylococcal phages using CRISPR-Cas10. ACS Synth Biol 6:2316–2325. doi:10.1021/acssynbio.7b00240. 28885820 PMC6087464

[8] Cater K, Dandu VS, Bari SMN, Lackey K, Everett GFK, Hatoum-Aslan A 2017 A novel *Staphylococcus* podophage encodes a unique lysin with unusual modular design. mSphere 2:e00040-17. doi:10.1128/mSphere.00040-17. 28357414 PMC5362749

